# Effects of atorvastatin in suppressing pulmonary vascular remodeling in rats with chronic obstructive pulmonary disease

**DOI:** 10.1016/j.clinsp.2023.100252

**Published:** 2023-07-15

**Authors:** YongHong He, SongPing Wang, Yuying Li, Jun Deng, Lan Huang

**Affiliations:** a Department of Respiratory and Critical Care Medicine, The Affiliated Hospital of Southwest Medical University, Luzhou City, Sichuan Province, China; b Department of Respiratory and Critical Care Medicine, Chengdu Second People's Hospital, Chengdu City, Sichuan Province, China

**Keywords:** Atorvastatin calcium, HDAC2, COPD rats, Pulmonary vascular remodeling

## Abstract

**Objective:**

To investigate the effects of atorvastatin calcium on pulmonary vascular remodeling, the authors explored the regulatory mechanism of Histone Deacetylation Enzyme-2 (HDAC2) in rats with Chronic Obstructive Pulmonary Disease (COPD), and provided a new direction for drug treatment in the progression of vascular remodeling.

**Methods:**

Eighteen female SD rats were randomly divided into control (Group S1), COPD (Group S2), and atorvastatin calcium + COPD (Group S3) groups. A COPD rat model was established by passive smoking and intratracheal injection of Lipopolysaccharide (LPS). Haematoxylin and eosin staining and Victoria Blue + Van Gibson staining were used to observe pathological changes in the lung tissue. The pulmonary vascular inflammation score was calculated, and the degree of pulmonary vascular remodeling was evaluated. The ratio of Muscular Arteries in lung tissue (MA%), the ratio of the vessel Wall Area to the vessel total area (WA%), and the ratio of the vessel Wall Thickness to the vascular outer diameter (WT%) were measured using imaging software. The expression of HDAC2 was measured using western blotting, ELISA (Enzyme-Linked Immunosorbent Assay), and qPCR (Real-time PCR).

**Results:**

Compared with the control group, the degree of pulmonary vascular inflammation and pulmonary vascular remodeling increased in rats with COPD. The WT%, WA%, and lung inflammation scores increased significantly; the expression of HDAC2 and HDAC2mRNA in the serum and lung tissue decreased, and the level of Vascular Endothelial Growth Factor (VEGF) in the lung tissues increased (*p* < 0.05). Compared with the COPD group, the lung tissues from rats in the atorvastatin group had fewer inflammatory cells, and the vascular pathological changes were significantly relieved. The WT%, WA%, and lung inflammation scores decreased significantly; the expression of HDAC2 and HDAC2mRNA in the serum and lung tissues increased, and the level of VEGF in the lung tissues decreased (*p* < 0.05).

**Conclusion:**

The present study revealed that atorvastatin calcium could regulate the contents and expression of HDAC2 in serum and lung tissues and inhibit the production of VEGF, thereby regulating pulmonary vascular remodeling in a rat model with COPD.

## Highlights

• HMG-CoA reductase inhibitor statins can inhibit vascular remodeling.
• The regulation of HDAC2/NF-κB signaling pathway can improve pulmonary vascular remodeling.
• Atorvastatin can be used in the early intervention of pulmonary vascular remodeling.

## Introduction

Chronic Obstructive Pulmonary Disease (COPD) is a common public health concern and is mainly caused by the inhalation of harmful and toxic particles or gases. It causes abnormal development of bronchial airway and lung tissue alveoli, decreased pulmonary function, accompanied by restricted airflow, and a series of respiratory symptoms. Moreover, COPD affects the quality of public health and increases the medical, economic, and physiological burden on society, resulting in a higher death rate. According to the World Health Organisation statistics, COPD is currently among the top three chronic diseases in the world [1]. Inflammation of the airway and pulmonary parenchyma are the main features of COPD, which also causes further deterioration in pulmonary hypertension and remodeling of the airway and pulmonary vessels, leading to respiratory failure and pulmonary heart disease. Statins, lipid-regulating drugs, are not only used in the prevention and treatment of cardiovascular disease but also in medicine as anti-inflammatory and anti-oxidation drugs, which could improve endothelial function and regulate immunity and other effects [2,3]. Statins improve the clinical symptoms of patients with COPD, delay deterioration, and reduce mortality [4,5]. Therefore, this study aimed to explore whether atorvastatin calcium could improve pulmonary vascular remodeling through the expression of histone deacetylase 2 and provide a new theory for the treatment of COPD patients.

## Materials and methods

### Animals and reagents

This study was conducted in the Animal Experiment Centre of Southwest Medical University with animal certificate number: SYSK (Sichuan) 2020-021 and involved SPF-grade SD female rats (160±20g). All animals were exposed to alternate cycles of 12h of light and darkness at 26°‒28°C and 50%‒60% humidity and were provided ad libitum access to food and water. All animal experiments were approved and performed in accordance with the guidelines set forth by the Animal Care and Use Committee of Southwest Medical University. All experimental procedures complied with the Declaration of Helsinki of the World Medical Association. This study involved the following: Atorvastatin calcium (Lipitor, Pfizer Inc, National drug approval H20051408); Lipopolysaccharide (LPS) (Sigma, USA); cigarette (nicotine 1.1 mg, carbon monoxide 12 mg, China Tobacco industrial); Rat Histone Deacetylation Enzyme-2 (HDAC2) ELISA Kit (ELK Biotechnology); Western Blot kit (Hycell Biotechnology company); rabbit anti-rat HDAC2 monoclonal antibody; rabbit anti-rat Vascular Endothelial Growth Factor (VEGF) polyclonal antibody; internal reference GAPDH (reduced Glyceraldehyde-Phosphate Dehydrogenase) antibody and sheep anti-rabbit second antibody (ASPEN, USA); RNA extraction kit, reverse transcription and quantitative real-time PCR kit (ELK Biotechnology); Haematoxylin and Eosin (HE) stain (Sinophenol Group Chemical Reagent Co., LTD); Victoria blue and picric acid-acidic magenta dye solution (Aspen, USA); PCR instrument (Gene amplifying apparatus, TC XP, BIOER Technology, China).

### COPD rat model and treatment

After one week of acclimatization to laboratory conditions, all SD female rats were randomly divided into three groups: control (n = 6), COPD (n = 6), and atorvastatin intervention (n = 6). On days 1 and 14, the rats in the COPD and atorvastatin groups were challenged with passive smoking and repeatedly instilled with 200 μg of LPS in the airway. The control rats were injected with 200 μL saline as previously described; however, inhalation of cigarettes was not permitted. On days 2‒13 and days 15‒42, the rats were placed in a self-produced fumigating box for smoking (twice a day, at an interval of 6h, 10 cigarettes each time) [6,7], and the rats in the atorvastatin group were given atorvastatin (10 mg/kg.days) by daily gavage before smoke inhalation [8,9]. The control and atorvastatin groups were administered the same saline by gavage at the same time, and the date of model establishment was 42 days. The tar content was 12 mg per cigarette, and the concentration of smog was approximately 15% (v/v) within a box with five cigarette-burning cups. The control group did not undergo fumigation.

### Serum collection

All rats were fully anaesthetized with 3% pentobarbital sodium injection (1.5 mL/kg). After anesthesia, the chest was opened to draw blood from the heart, and then the blood was centrifuged at 3000 r/min for 15 min. Subsequently, the serum supernatant fluids were extracted and deposited into tubes and placed in a -80°C low-temperature refrigerator.

### Preparation of tissue and histological examination

The upper lobe of the right lung was excised and washed in normal saline to remove blood stains, fixed with 4% paraformaldehyde, embedded in paraffin, and stained with HE. Histopathological changes were observed by light microscopy, and the degree of pulmonary vascular inflammation was evaluated as follows:

1. No inflammatory cells were observed in light microscopy (0 points).
2. A few inflammatory cells infiltrate the lung tissues (1 point).
3. Inflammatory cell infiltration was obvious in the lung tissues but was not evenly distributed (2 points).
4. Inflammatory cell infiltration was obvious and evenly distributed in the lung tissues (3 points).
5. Several inflammatory cells were evenly distributed and aggregated (4 points).

The middle lobe of the right lung was treated in the same way as that of the upper lobe of the right lung and stained with Victoria blue. The image analysis software was used to determine the index of pulmonary vascular remodeling, and a muscle artery of 50‒100 µm was selected for image analysis at 400 × magnification. The ratio of the vessel Wall Area to the vessel total area (WA%) and the ratio of the vessel Wall Thickness to the vascular outer diameter (WT%) were calculated. For quantitative analysis, microscopic images of lung tissue sections were analyzed using Image-Pro Plus v.6.0 (Media Cybernetics, Inc.). Pulmonary vascular remodeling in the arterioles was evaluated using the percentage of vascular Wall Thickness (WT%) and the percentage of the vascular Wall Area (WA%). The formula for WT% was WT% = [2 × (external diameter of the pulmonary arterioles - internal diameter of the pulmonary arterioles) ]./(external diameter of the pulmonary arterioles) × 100. The formula for WA% was WA% = (external area - internal area)/external area × 100 [10].

The lower part of the right lung tissue was perfused with ice-cold heparinized saline, isolated, stored at -80°C until analysis, and used for western blotting and real-time PCR.

### ELISA for determining HDAC2 in serum

The serum samples collected from the three groups were immediately separated by centrifugation. According to the instructions of the ELISA kit, the double antigen sandwich method was adopted: 100 μL of standard or test sample was added and incubated at 37°C for 2h; 100 μL of biotinylated anti-rat HDAC2 antibody diluent was added (reacted at 37°C for 1h) and washed in the PBS thrice. Overall, 100 μL of streptavidin-HRP working solution was added to each well. The wells were covered with a plate sealer and incubated for 1h at 37°C. These steps were repeated five times. In total, 90 μL of TMB substrate solution was added to each well and incubated at 37°C for 20 min, and 50 μL of stop reagent was added to each well. The insertion order of the stop reagent should be the same as that of the TMB substrate solution, following which the microplate reader should be used, and measurements should be taken immediately at 450 nm. Optical density and HDAC2 levels were calculated based on the standard curve.

### Western blot for analyzing the level of VEGF and HDAC2

The upper lobes of the left lung were collected for protein extraction, and protein concentrations were determined using a BCA protein assay kit, SDS-PAGE (sodium dodecyl sulfate-polyacrylamide gel electrophoresis) electrophoresis, membrane transfer, and sealing. The membranes were incubated at 4°C overnight with primary antibodies against HDAC2 (1:1000 dilution), VEGF (1:1000 dilution), and GAPDH (1:1000 dilution); GADPH was used as a control. The membranes were washed five times with TBST (Tris-buffered saline Tween) and incubated with secondary antibodies (1:1000 dilution) at room temperature for 1h. The films were scanned after exposure and were developed and fixed. The film was prepared using a gel image processing system, and a semi-quantitative analysis was performed.

### Real-time PCR

The middle lobes of the lung were used for total RNA extraction. RNA was reverse transcribed into cDNA, and qPCR amplification was performed. The primers used for the target genes are listed in Table 1. The initial activation was at 95°C for 3 min, 60°C for 30s, 95°C for 15s, 60°C for 1 min, 95°C for 15s, and 60°C for 30s for 40 cycles. The relative expression of HDAC2 was calculated by 2^−ΔΔCt^ method.

**Table 1:** Primer sequence for QPCR.

**Gene**	**Primer sequence (5′→3′)**	**Value**
HDAC2	Forward: ACCTAACTGTCAAAGGTCACGCT	152 bp
	Reverse: ACCTAACTGTCAAAGGTCACGCT	
GAPDH	Forward: GCCAAGGTCATCCATGACAAC	152 bp
	Reverse: GTGGATGCAGGGATGATGTTC	

### Statistical analysis

Statistical analyses were performed using SPSS 19.0, and the data are presented as mean ± SEM (*X*±*S*). One-way analysis of variance followed by an S–N–K test was used to determine significant differences between the two groups; *p* < 0.05 was defined to be statistically significant.

## Results

### Histopathological features of lung tissues

#### HE

**Fig. 1:**
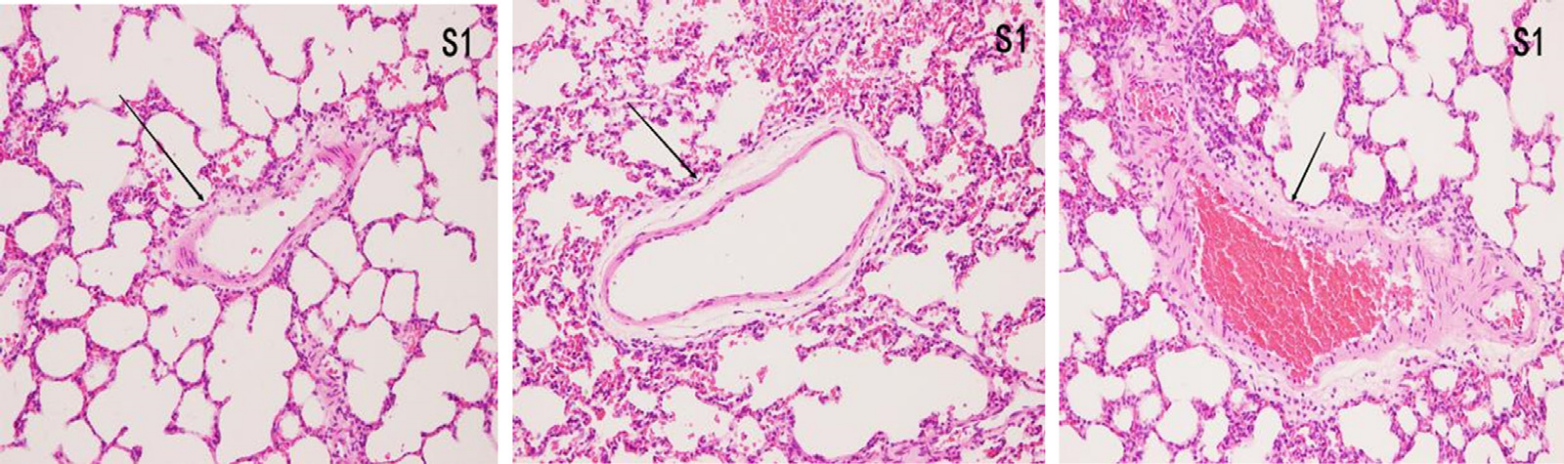
Pulmonary airway and vascular changes in the control group (× 200).

**Fig. 2:**
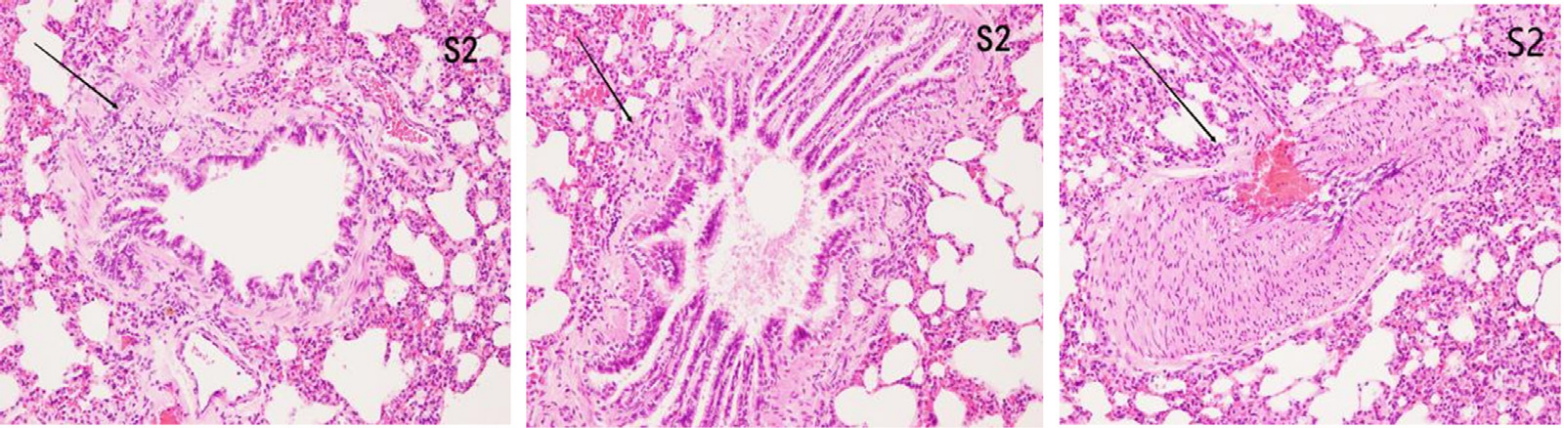
Pulmonary airway and blood vessel changes in the COPD group (× 200).

**Fig. 3:**
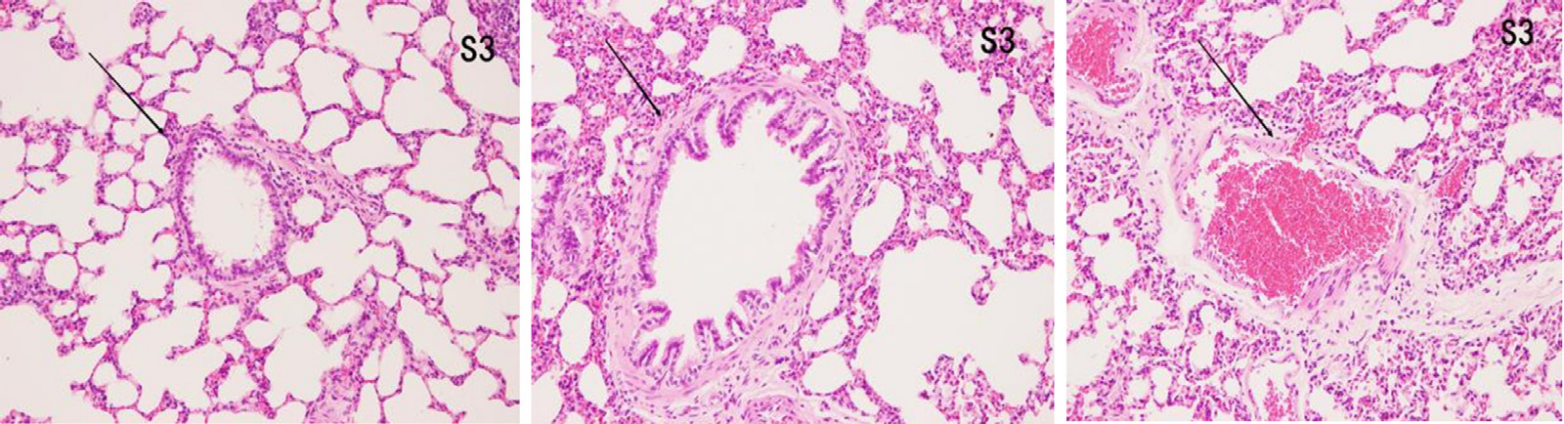
Pulmonary airway and vascular changes in the atorvastatin group (× 200).

In the control group, there was no damage or inflammation of the pulmonary vessels and alveolar structure, and the range of the inflammation score was 0‒1, with a lower inflammation score. In the COPD group, many inflammatory cells gathered in the lung tissue, the alveolar septa were markedly oedematous, and the pulmonary vessels were thickened. Infiltration of inflammatory cells was observed, the partial alveolar septal rupture was present, and the inflammation score was high. Compared to the COPD group, the infiltration of inflammatory cells in the atorvastatin calcium group was significantly reduced. The degree of pulmonary vascular smooth muscle hyperplasia was lower, with less damage to the normal lung tissue structure and a significantly lower lung histopathological score. (Fig. 1, Fig. 2, Fig. 3, Table 2).

**Table 2:** Changes of pulmonary vascular remodeling indicators in each group (inflammation score, WA%, WT%, and MA% in lung tissues).

**Group**	**n**	**Inflammation score**	**WA%**	**WT%**	**MA%**
Control (S1)	6	0.54±0.07	27.25±1.02	16.27±0.44	35.63±1.26
COPD (S2)	6	2.63±0.45*	40.70±1.14^a^	18.37±0.38^a^	59.26±1.49^a^
Atorvastatin(S3)	6	1.96±0.36^a^^,^^b^	30.41±0.87^a^^,^^b^	17.22±0.20^a^^,^^b^	48.54±1.52^a^^,^^b^
*F*		30.09	284.98	50.95	418.33
*P*		0.001	<0.001	<0.001	<0.001

Compared with the control group.

a *p* < 0.05; compared with COPD group.

b *p* < 0.05.

#### Victoria blue and van gibson

**Fig. 4:**
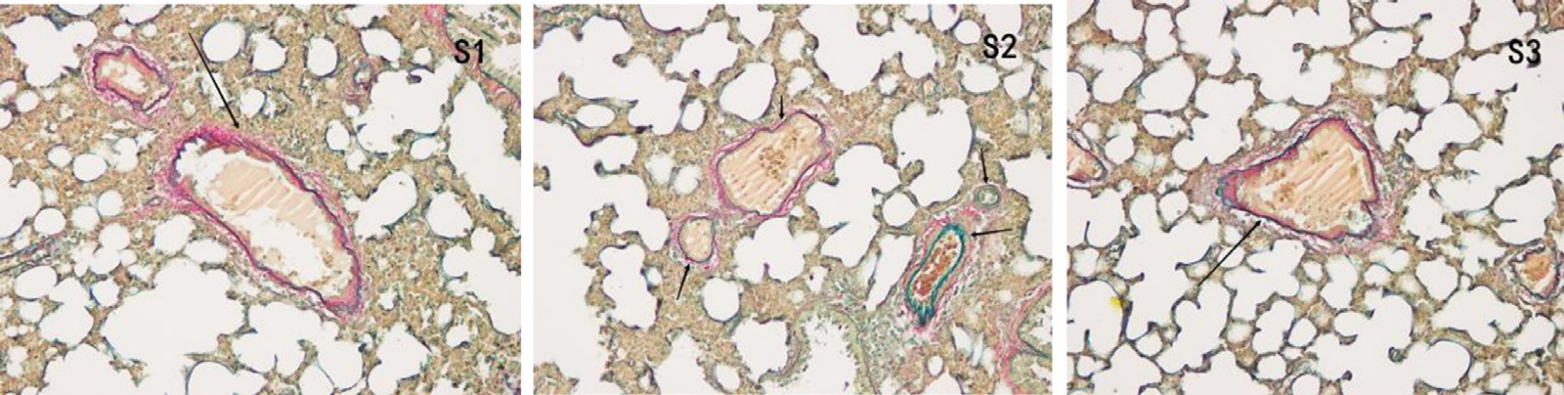
Pulmonary vascular changes in the control, COPD, and atorvastatin groups (Victoria blue + VG staining × 200).

In the control group, all arterial walls in the lung tissue were normal. In the COPD group, there were intact elastic plates around the arteries with significantly induced muscularization. Stenosis and occlusion of the arterioles were observed in the lungs, which was accompanied by inflammatory cell infiltration around the vessel wall and lung tissues. WA% and WT% were significantly increased (*p* < 0.05). Compared to the COPD group, the degree of pulmonary vascular remodeling was significantly reduced in the atorvastatin group. WA% and WT% of the pulmonary vessels decreased (*p* < 0.05) (Fig. 4).

#### ELISA

**Fig. 5:**
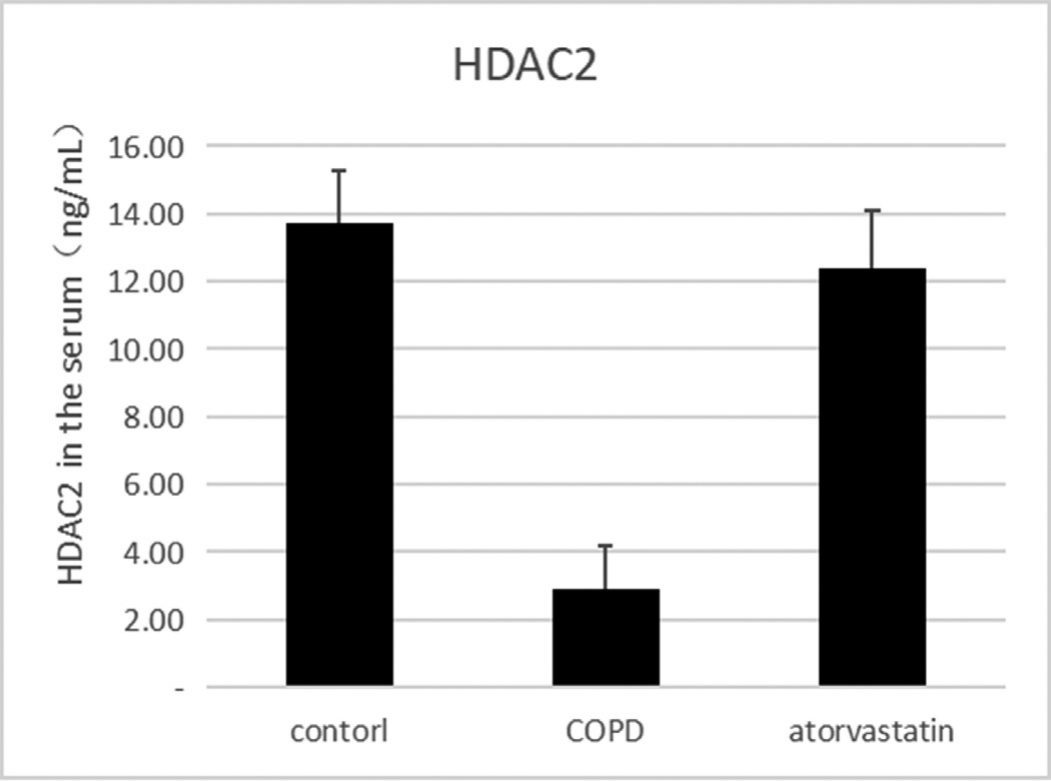
Concentration of HDAC2 in serum was measured using ELISA.

The effects of atorvastatin on cytokine production in the serum of COPD rats and the concentration of HDAC2 in the serum were measured using ELISA. The serum concentration of HDAC2 in the COPD group was significantly decreased than in the control group (*p* < 0.05). The serum level of HDAC2 in the atorvastatin group was significantly increased than in the COPD group (*p* < 0.05) (Fig. 5, Table 3).

**Table 3:** Comparison of HDAC2 and VEGF expression levels in the serum and lung tissues of rats between the three groups.

			**Lung tissue**			
**Group**	**n**	**HDAC2 in serum (μg/L)**	**HDAC2 protein**	**HDAC2 mRNA**	**VEGF protein**	**VEGF mRNA**
Control (S1)	6	13.794±1.549	0.356±0.056	1.273±0.177	0.121±0.003	1.21±0.107
COPD (S2)	6	2.921±1.288^a^	0.102±0.016^a^	0.292±0.022^a^	0.269±0.011^a^	4.03±0.442^a^
Atorvastatin (S3)	6	12.135±1.622^a^^,^^b^	0.313±0.028^a^^,^^b^	0.883±0.079^a^^,^^b^	0.139±0.005^a^^,^^b^	1.43±0.13^a^^,^^b^
F		184.472*	77.384*	114.502*	690.386*	102.86*

⁎ *p* < 0.01.

a Compared with the control group, *p* < 0.05.

b Compared with the COPD group, *p* < 0.05.

#### Western blot

**Fig. 6:**
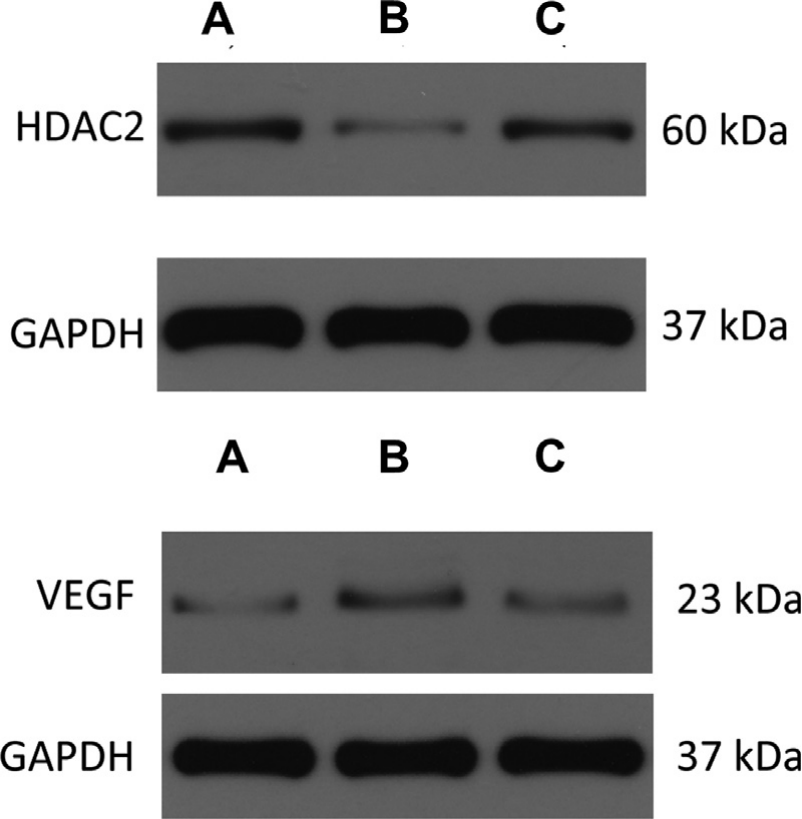
Expression of HDAC2 and VEGF in the lung tissue. (A) Control group; (B) COPD group; (C) Atorvastatin group.

**Fig. 7:**
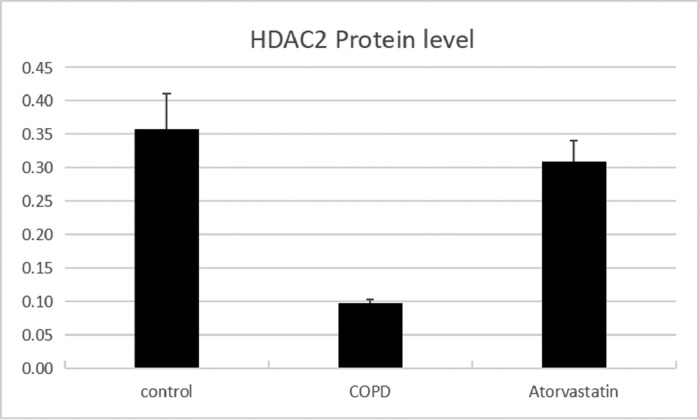
Concentration of HDAC2 in lung tissues.

**Fig. 8:**
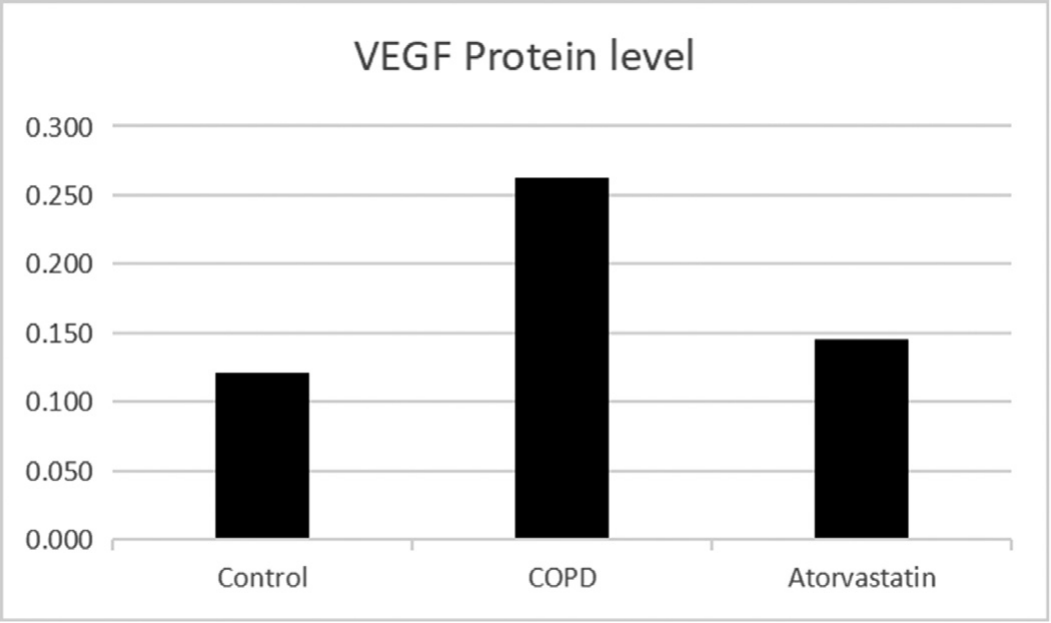
Concentration of VEGF in lung tissues.

The expressions of HDAC2 and VEGF in the lung tissue of the three groups were measured by western blotting (Fig. 6, Fig. 7, Fig. 8, Table 3). The analysis indicated that the protein expression of HDAC2 was decreased in the COPD group compared with the control group (*p* < 0.05). The expression of HDAC2 was increased in the atorvastatin group than in the COPD group (*p* < 0.05). The western blot analysis suggested that the protein expression of VEGF was increased in the COPD group than in the control group (*p* < 0.05). The level of VEGF was decreased in the atorvastatin group than in the COPD group (*p* < 0.05).

#### Real-time PCR

**Fig. 9:**
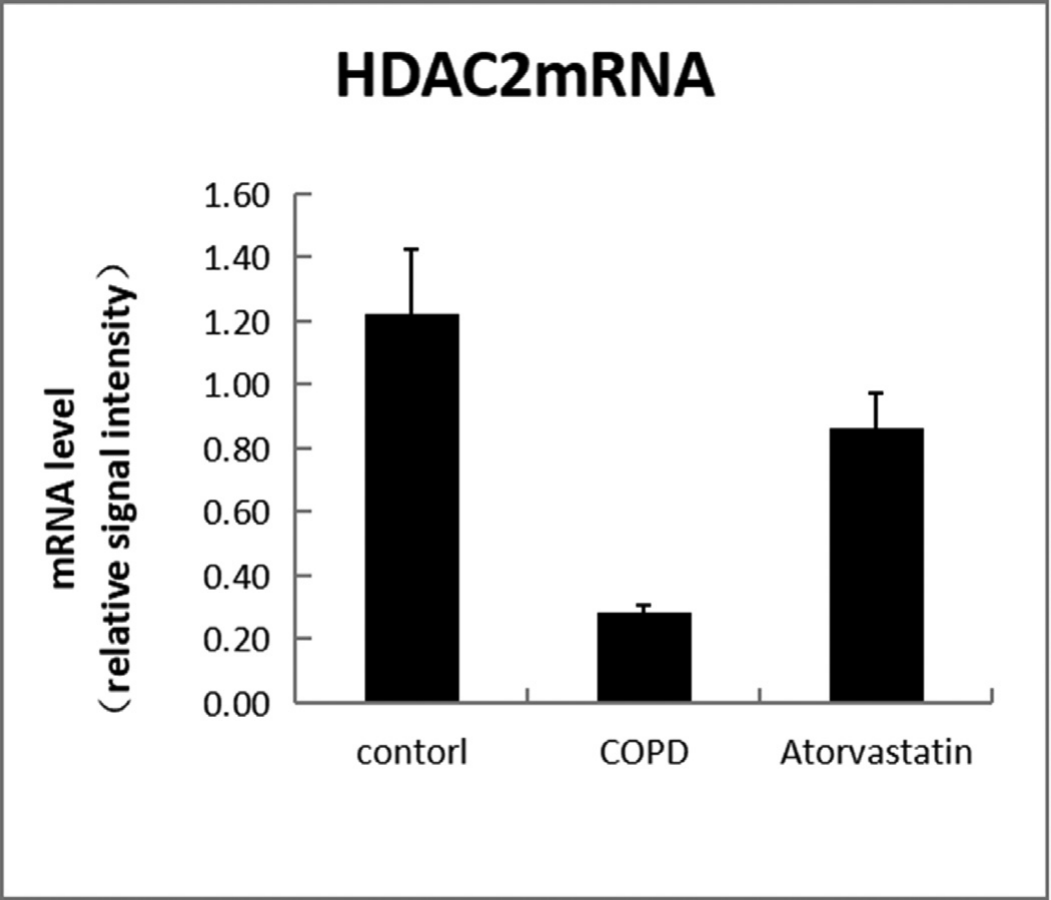
Level of HDAC2Mrna.

**Fig. 10:**
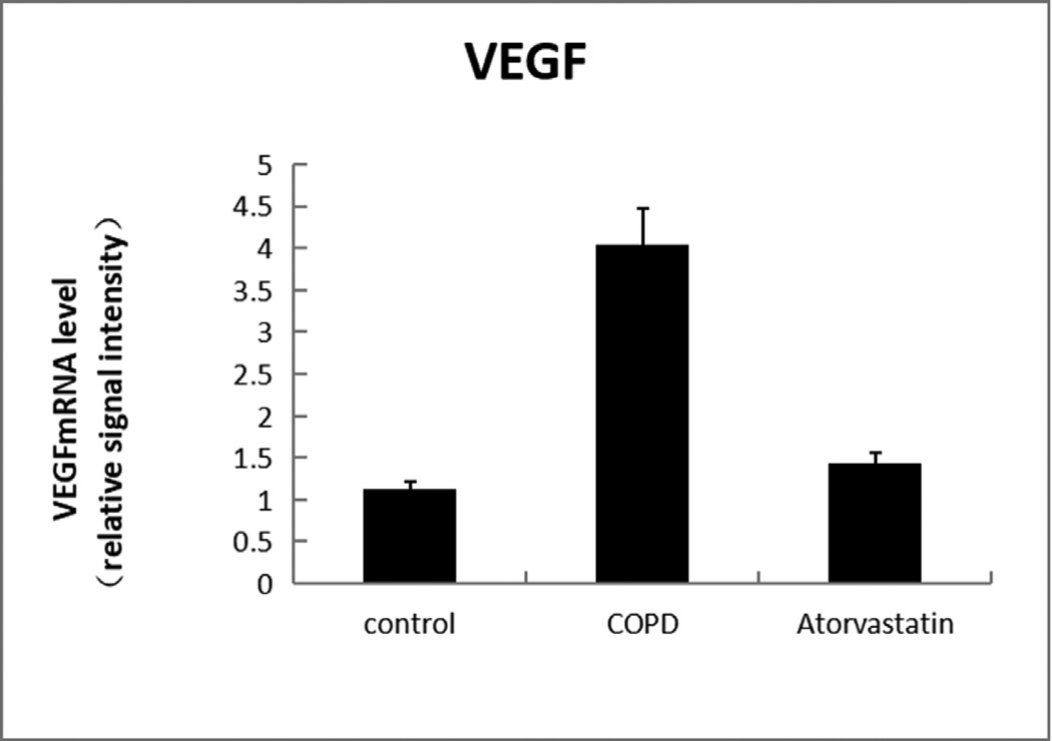
Level of VEGFmRNA.

In order to assess whether atorvastatin can influence the expression and activity of HADC2 and VEGF, the transcription of HDAC2 and VEGF genes in the lung tissue was also analyzed by real-time PCR. The levels of HDAC2mRNA were decreased and those of VEGFmRNA were increased in the COPD group than in the control group (*p* < 0.05). The levels of HDAC2mRNA were increased and those of VEGFmRNA were decreased in the atorvastatin group than in the COPD group (*p* < 0.05) (Figs. 9,10; Table 3).

## Discussion

COPD is a common chronic respiratory disease that greatly affects the health of individuals. It is caused by long-term cigarette exposure and inhalation of toxic substances, which damage the airway structure; stimulate the production of a large number of inflammatory cells; increase airway mucus secretions, vascular permeability, and edema; and promote the proliferation of tracheal vascular smooth muscle. However, in COPD patients, compared with the airway remodeling changes caused by inflammation and hypoxia, the pathological and physiological changes in pulmonary vessels caused by inflammatory pulmonary hypertension are often accompanied or even preceded [11]. With the development of the erosion of pulmonary vessel inflammation, the structural changes of pulmonary arterioles, influences of pulmonary blood circulation, formation of arterial high pressure, and pulmonary vascular remodeling occur. The mechanism for this is as follows: 1) Changes in the cellular ion channels owing to chronic and repeated cough and sputum production result in a sharp decline in the pulmonary function of patients with COPD. Dyspnoea leads to severe hypoxia, accompanied by significant inflammatory changes that force K+ in pulmonary vascular smooth muscle cells. The membrane potential of the Ca+ ion channels change and is unable to depolarise normally. With the increase in ATP consumption in cells, the normal opening and closing function of ion channels is disturbed, and the constriction of pulmonary vessels is seriously affected [12]. 2) Protein Kinase C (PKC) signaling is mainly responsible for the transduction of extracellular information. In extreme ischemia and hypoxia, the phospholipase in membrane receptors on the PKC-linked pathway is rapidly activated, resulting in cascade reactions and the phosphorylation of partial residues of NOS serine. The endothelium-derived relaxing factor is reduced with the loss of normal relaxation of a large number of vessels, and pulmonary vascular resistance is further increased [13]. 3) Hypoxia leads to NO release; hence, the development of vascular autonomic dysfunction slows or even stops the repair of injured endothelial cells, accelerates the release of apoptosis and vascular proliferative factors, including detached VEGF and ET-1, and decreases the contraction and relaxation function of the pulmonary artery, thus resulting in vascular reconstruction [14]. In pulmonary vascular remodeling, vascular smooth muscle is thickened owing to inflammation, hypoxia, and other injuries. The three layers of the inner, middle, and outer membranes of the pulmonary arterioles are thickened to varying degrees. With the appearance of dyed internal and external elastic plates, the proportion of muscular arteries is significantly increased. Therefore, combined with relevant literature, the increased proportion of muscular arteries can be regarded as one of the main indicators of pulmonary vascular remodeling [15].

HDAC is a conventional structural enzyme of the chromosome that primarily targets the expression of inflammatory genes [16]. Among the HDAC family members, HDAC2, which is mainly located in pulmonary alveolar cells and pulmonary vascular endothelial cells, plays a decisive role in the expression of inflammatory genes in lung tissue. Some studies have illustrated that HDAC2 is considered the main family member of HDAC that is involved in the pathogenesis of COPD, thus inhibiting the expression of inflammatory genes, blocking the release of anti-inflammatory factors, and determining the severity of airway inflammation, with important effects on the degree of airflow limitation of COPD airway [17,18]. During the progression of COPD, as the expression of HDAC2 gradually decreases, the chromatin cofactors are unable to assist chromatin and tightly coil the transcription RORγ T, which cannot complete acetylation, and competitively increase the nuclear factor NF-κB acetylation. A large number of inflammatory factors such as IL-8, IL-17, Tumor Necrosis Factor (TNF)-α, and vascular endothelial factors such as VEGF and ET-1 promote pulmonary vascular endothelial function disorder and pulmonary vasoconstriction and cause the development of pulmonary vascular remodeling [19]. Atorvastatin is commonly used to lower cholesterol levels in clinical treatments by inhibiting 3-hydroxy-3-methylglutaryl coenzyme A, which catalyzes the rate-limiting step in the mevalonate biosynthesis pathway and is a key intermediate in cholesterol metabolism. Thus, it reduces the rates of stroke, myocardial infarction, vascular death, cerebrovascular events, and mortality from coronary artery disease [20]. Atorvastatin has also been the subject of extensive research to explore its potential beneficial effects on COPD. In addition to the lipid-lowering effect, atorvastatin could provide greater protection, than predicted, against cholesterol-lowering effects, including anti-inflammatory, antioxidant, immunomodulatory effects, improved endothelial cell function, and anti-thrombotic actions [21]. These pleiotropic statin-induced protective effects could be used to manage chronic lung diseases with inflammatory and oxidative stress components, such as asthma, COPD, and pulmonary hypertension [22].

Herein, the aggregation of inflammatory cells around the pulmonary vessels in the atorvastatin group was reduced than in those in the COPD group. The degree of vascular endothelial injury and pulmonary vascular inflammation was lower. The ratio of the vessel Wall Area and vessel total area (WA%), the ratio of the vessel Wall Thickness and vascular outer diameter (WT%), and the expression of VEGF in the lung tissue were significantly decreased. This indicated that atorvastatin could alleviate vascular endothelial injury, pulmonary vascular inflammation, and remodeling to a certain extent. The levels of HDAC2 in serum and lung tissue were significantly decreased in the COPD group. Owing to atorvastatin intervention, the expression of HDAC2 in the atorvastatin group was significantly increased, with a lower degree of inflammatory response and pulmonary vascular remodeling. According to the results of this study and the literature, the underlying mechanism for this might be that in the tricarboxylic acid cycle, statins prevent acetyl-CoA from producing cholesterol; reduce the production of guanosine triphosphate binding protein; block NF-κB from entering the nucleus; inhibit the release of IL-8, TNF-α, and other inflammatory factors; and terminate the development of inflammation. Atorvastatin calcium can also significantly improve pulmonary vascular inflammation and regulate the degree of pulmonary vascular remodeling. Therefore, combined with the experimental results and reference to relevant literature [23], the mechanism may be that atorvastatin inhibits the production of acetyl-coa, reduces the content of GTP, and inhibits NF-κB, thereby competitively reducing the specific binding of HDAC2, increasing the acetylation of NF-κB, and reducing the release of inflammatory factors and inflammatory mediators such that pulmonary vascular inflammation is significantly reduced. It can improve vascular permeability and vasodilator function, reduce the migration of eosinophils and lymphocytes, reduce the entry of inflammatory factors into the tissue, reduce vascular permeability, downregulate pulmonary vascular endothelial factors and other related inflammatory mediators, and prevent vascular inflammation caused by airway cell chemotaxis. This is consistent with the findings of previous studies [24,25]. In addition, atorvastatin improves the function of vascular endothelial cells, maintains vascular permeability and vasomotor function, and stabilizes the pulmonary intravascular environment. Based on this study, atorvastatin inhibited the expression of VEGF in lung tissue, which confirmed that atorvastatin prevented pulmonary vascular hyperplasia and essentially alleviated the degree of pulmonary vascular remodeling. This mechanism might be related to the fact that atorvastatin promotes an increase in HDAC2 content and reduces the production of VEGF. It is unclear whether atorvastatin directly inhibits the release of VEGF in vascular endothelial cells; this requires further investigation.

The present study had some limitations. First, the sample size was not sufficient. Sufficient sample size can further expand the accuracy of experimental research, reduce experimental bias caused by experimental operations, and reduce experimental errors. Second, in reference to the literature and studies on the establishment of the classic COPD model, the detection of pulmonary function was not listed as a necessary condition for the successful preparation of the COPD rat model. Moreover, in this experiment, owing to the obvious difference in the lung function of rats in each group, the experimental error bias was large. Third, after referring to the results of several experiments and relevant literature, this study used a single dose of atorvastatin for all groups of rats. However, there is no relevant literature report that reveals conclusive evidence on whether atorvastatin calcium tablets are administered at the appropriate dosage and frequency for the treatment of COPD in a rat model, which needs to be further improved. Fourth, atorvastatin improved the expression of HDAC2 and VEGF in this study; however, the mechanism remains unclear whether atorvastatin directly acts on the expression of VEGF to treat COPD pulmonary vascular remodeling or indirectly controls the production of VEGF by regulating the concentration of HDAC2. At present, there is neither an accurate report nor literature to discuss this part of the mechanism nor there is a complete signal pathway to explain this phenomenon; therefore, further experiments are needed to confirm the relevant principles.

In conclusion, the research conducted by the authors demonstrated that atorvastatin can improve pulmonary vascular inflammation and pulmonary vascular remodeling in COPD to a certain extent, possibly by increasing the expression level of HDAC2, inhibiting the expression of inflammation-related genes, and reducing the release of the inflammatory factor pulmonary vascular endothelial factor.

## Ethical approval and consent to participate

This study was approved by the Research Ethics Committee and the Animal Use Ethics Committee of The Affiliated Hospital of Southwest Medical University Fed under number 20220921-003, This study conformed to The ARRIVE Guidelines Checklist for studies on animals.

## Declaration of Competing Interest

The authors declare no conflicts of interest.
